# Exercise Training and Cardiac Rehabilitation in COVID-19 Patients with Cardiovascular Complications: State of Art

**DOI:** 10.3390/life11030259

**Published:** 2021-03-21

**Authors:** Mariaconsiglia Calabrese, Marina Garofano, Roberta Palumbo, Paola Di Pietro, Carmine Izzo, Antonio Damato, Eleonora Venturini, Severino Iesu, Nicola Virtuoso, Andrea Strianese, Michele Ciccarelli, Gennaro Galasso, Carmine Vecchione

**Affiliations:** 1 Rehabilitation Department, A.O.U. “San Giovanni di Dio e Ruggi d’Aragona”, 84125 Salerno, Italy; macalabrese@unisa.it; 2 Department of Medicine, Surgery and Dentistry, University of Salerno, Via S. Allende 43, 84081 Baronissi, Italy; pdipietro@unisa.it (P.D.P.); carmine.izzo93@gmail.com (C.I.); n1virtuoso@gmail.com (N.V.); andreastrianese@hotmail.com (A.S.); mciccarelli@unisa.it (M.C.); ggalasso@unisa.it (G.G.); 3 Associazione Italiana Fisioterapia (A.I.FI.) sezione territoriale Campania, Via Pinerolo 3, 00182 Roma, Italy; marinag90@inwind.it (M.G.); r.robertapalumbo@gmail.com (R.P.); 4 Vascular Physiopathology Unit, Department of Angio-Cardio-Neurology, IRCCS Neuromed, 86077 Pozzilli, Italy; antonio.damato85@gmail.com (A.D.); eleonora.venturini@neuromed.it (E.V.); 5 Heart Department, University Hospital “San Giovanni di Dio e Ruggi d’Aragona”, 84125 Salerno, Italy; severino.iesu@sangiovannieruggi.it

**Keywords:** COVID-19, rehabilitation, cardiovascular

## Abstract

Recent scientific literature has investigated the cardiovascular implications of COVID-19. The mechanisms of cardiovascular damage seem to involve the protein angiotensin-converting enzyme 2 (ACE2), to which severe acute respiratory syndrome (SARS) coronavirus-2 (CoV-2) binds to penetrate cells and other mechanisms, most of which are still under study. Cardiovascular sequelae of COVID-19 include heart failure, cardiomyopathy, acute coronary syndrome, arrhythmias, and venous thromboembolism. This article aims to collect scientific evidence by exploiting PubMed, Scopus, and Pedro databases to highlight the cardiovascular complications of COVID-19 and to define the physiotherapy treatment recommended for these patients. Exercise training (ET), an important part of cardiac rehabilitation, is a powerful tool in physiotherapy, capable of inducing significant changes in the cardiovascular system and functional in the recovery of endothelial dysfunction and for the containment of thromboembolic complications. In conclusion, due to the wide variety of possible exercise programs that can be obtained by combining intensity, duration, and speed in various ways, and by adjusting the program based on continuous patient monitoring, exercise training is well suited to the treatment of post-COVID patients with an impaired cardiovascular system of various degrees.

## 1. Introduction

Coronavirus disease 2019 (COVID-19) is a contagious disease caused by severe acute respiratory syndrome coronavirus-2 (SARS-CoV-2). COVID-19 has reached pandemic status and has overwhelmed health care systems, devastated the global economy, and severely restricted everyday life. In this time of crisis, the medical and scientific communities have gathered to understand as much as possible about this disease. Great advances have been made. Knowledge about its pathogenesis, clinical manifestation, preventive care, and therapeutic strategies has grown rapidly [1,2,3,4]. 

COVID-19 involves systemic inflammation with an increase in the oxidation state to varying degrees, depending on the severity of the symptoms accompanying the disease [5]. The systemic inflammatory state persists over time, defining what the scientific literature today describes as “long COVID“ [6].

Physical exercise, correctly structured and guided or supervised, intervenes in this inflammatory state by promoting the recovery of the antioxidant defenses [7].

Various hypotheses have been put forward regarding the mechanisms of damage at the cardiovascular level, and the most recognized seems to be associated with the transmembrane protein angiotensin-converting enzyme 2 (ACE2) [8,9]. 

The life cycle of SARS-CoV-2 begins with viral binding to cells via the membrane-bound glycoprotein angiotensin-converting enzyme 2 (ACE2) [10]. Once bound to ACE2, the virus is internalized via endocytosis [11]. The next step is membrane fusion, where the viral RNA genome enters the intracellular compartment, to be translated. The interaction between the encoded proteins and the viral RNA on the membrane of the endoplasmic reticulum and the Golgi apparatus results in viral budding and exocytosis [11,12,13].

**Figure 1:**
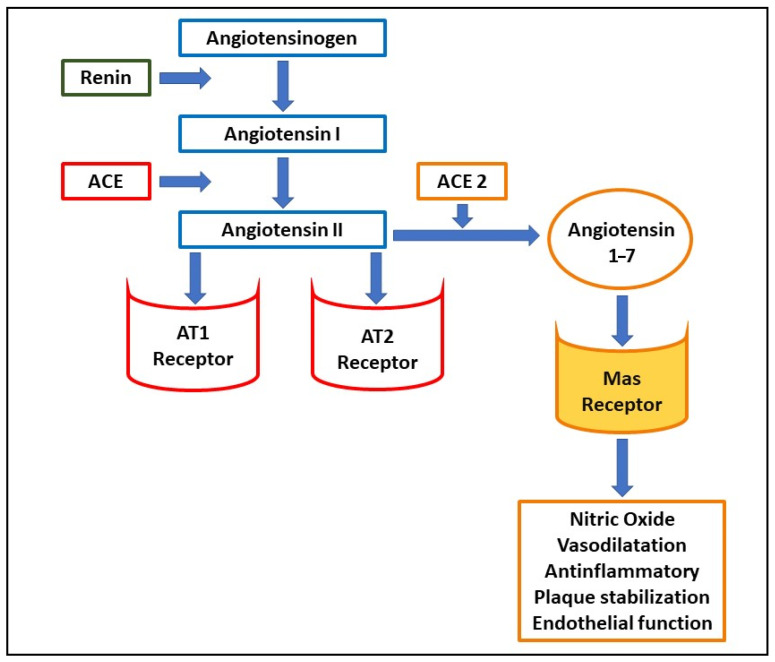
Schematic representation of the renin–angiotensin system and the function of the Mas receptor. Abbreviations: AT1, angiotensin II type 1 receptor; AT2, angiotensin II type 2 receptor; ACE, angiotensin-converting enzyme; ACE2, angiotensin-converting enzyme 2.

SARS-CoV-2 employs ACE2 as a receptor [10,14,15,16] and ACE2 carries out important functions in the cardiovascular system and cardiovascular pharmacology. This surface enzyme is widely expressed in lung tissue [17], in cardiovascular tissue [18] including the endothelia [19], renal, and intestinal tissue [20,21]. Once the virus has penetrated these tissues, it generates multiple damages, probably related to the inhibition of the protective pathways activated by ACE2 [17,22]. Physiologically, ACE2 constitutes a counter-regulator of the renin–angiotensin–aldosterone system, transforming angiotensin II (Ang II) into angiotensin 1–7 (Ang 1–7) (Figure 1). The latter, by binding to a specific Mas receptor, causes a reduction in blood pressure by vasodilatation and by increasing diuresis. In addition, Ang 1–7 carries out the endothelial protective activity by increasing the production of nitric oxide (NO), thus reducing vascular inflammation [23,24], and increasing the stability of the atherosclerotic plaques [25,26]. On the other hand, the inhibition of ACE2, causes an increase in angiotensin II, with its hypertensive and pro-oxidant effects [24,27,28]. However, the role of ACE2 seems to be controversial as according to recent studies, the soluble form of the receptor could have a protective role against coronavirus [29]. 

Hence, the prevalence of COVID-19 severe illness among patients with cardiovascular comorbidities has drawn much attention to ACE2 [30]. So much so, that consensus statements on the use of ACE inhibitors and angiotensin receptor blockers (ARBs) have been issued by major clinical societies [31,32]. 

Other mechanisms implicated in the damage to myocardial cells are:

- The cytokine storm generated by an unbalanced response by type 1 and type 2 helper T cells [33,34,35]
- The altered relationship between demand and supply of oxygen by the myocardium, originating from an increase in cardio-metabolic demand associated with systemic infection, not satisfied by diffusive respiratory deficit hypoxia and oxidative stress [22,36,37,38]
- Destabilization of atherosclerotic plaques [39,40].

In this article, we aimed to address the main cardiovascular knowledge and complications of COVID-19 in order to conceptualize potential strategies to rehabilitate patients with these kinds of after effects.

## 2. Cardiovascular Manifestations 

Cardiac injury has been reported in many studies as an important COVID-19 manifestation. Acute cardiac injury, in the studies to date, was defined in various ways including troponin elevation, electrocardiographic or echocardiographic abnormalities [1,39,41]. In hospitalized patients, the rate of COVID-19 cardiac involvement ranged between 7–28%. This percentage is largely dependent on the definition used and the severity of the single cases [1,39,41,42]. COVID-19 hospitalized patients with cardiac injury had worse outcomes with higher intensive care unit admission and death [1,39,41,42]. There have been reports of early cardiac injury in the absence of respiratory symptoms [43]. Troponin elevation was correlated to a higher mortality rate [44]. Overall, understanding the mechanisms underlying COVID-19 cardiac injury is necessary to implement and conceptualize possible cardiac rehabilitation protocols. The different complications may require different protocols and precautions.

### 2.1. Heart Failure and Myocarditis 

Heart failure and myocardial dysfunction occur in 10–52% of patients hospitalized for COVID-19 [42,43,45]. This percentage increases dramatically in patients with concomitant cardiac disease [46]. It is unclear whether heart failure is more commonly due to exacerbation of pre-existing left ventricular dysfunction than new cardiomyopathy (due to myocarditis or stress cardiomyopathy) [47,48]. The same goes for the incidence of systolic dysfunction and cardiogenic shock [49]. Right heart failure and associated pulmonary hypertension should also be considered, particularly in the context of severe parenchymal lung disease and acute respiratory distress syndrome (ARDS) [50]. 

Myocarditis has been widely reported and affects about 8–12% of hospitalized COVID-19 patients [1,3]. The presence of acute myocardial damage constitutes a negative prognostic factor for patient survival [36]. Damage mechanisms involved are viral myocarditis, demonstrated by autopsy tests that have detected the presence of viral ribonucleic acid (RNA) within myocardial cells, and systemic inflammation [50]. Due to the unavailability of cardiovascular magnetic resonance (CMR), it is often impossible to distinguish myocarditis from stress-induced cardiomyopathy or a myocardial cytokine release syndrome. Takotsubo and reverse Takotsubo stress cardiomyopathy have been reported in patients with COVID-19, characterized by mid-left ventricular or basal-to-mid left ventricle hypokinesis [51]. Moreover, COVID-19 can also mediate the intense release of pro-inflammatory cytokines with a consequent subacute depression of myocardial function [52,53]. Cytokine storm in COVID-19 patients is mediated mainly by increased TNF-alpha and interleukin plasma levels through neural sphingomyelinase, which blunts NO and beta-adrenergic signaling [54,55,56]. 

### 2.2. Acute Coronary Syndrome

Type 1 myocardial infarction generated by rupture of a plaque with thrombus formation may accelerate in patients with COVID-19 due to the presence of circulating cytokines, systemic inflammatory status, and the reduction of ACE2 expression, and therefore, the increased expression of angiotensin II [54,55]. In COVID-19 patients, due to reduction in pulmonary diffusion, a type 2 infarction is also possible, caused by hypoxia related to the inadequate relationship between the demand and supply of oxygen by the myocardial cells [54]. In COVID-19 patients there is also evidence of myocardial injury (between 4.2% and 25% due to disseminated intravascular coagulation) [57,58,59]. This eventuality is associated with multiorgan failure through thrombosis related reduction in perfusion, and finally, in bleeding [60,61]. Thrombosis of coronary arteries due to disseminated intravascular coagulation (DIC) equally causes focal necrosis of the myocardium with possible severe cardiac dysfunction [62].

### 2.3. Arrhythmias

Arrhythmia is a common manifestation found in patients with COVID-19, including 7.3% to 44% of patients admitted to intensive care [63,64]. On the specific type of arrhythmia, a case study of 187 hospitalized patients showed sustained ventricular tachycardia or ventricular fibrillation in 5.9% of the patients [1,39,41]. Atrial fibrillation is the most common arrhythmia in COVID-19 patients; the incidence is particularly high in the acute phase [65,66]. Patients with prior atrial fibrillation show a worsening of its management [67]. The high prevalence of arrhythmia could be, in part, attributable to metabolic problems, hypoxia, neurohormonal or inflammatory stress, however, new onset of malignant tachyarrhythmias accompanied by an increase in troponin should raise suspicion of underlying myocarditis [68,69].

### 2.4. Venous Thromboembolism

Research shows that COVID-19 patients are at higher risk for venous thromboembolism (VTE) and pulmonary embolism (PE). In fact, evidence shows an increase in D-Dimer levels, an important coagulation parameter [1,3,39,41]. The criteria for DIC had been found in 71.4% of patients after death [63,70]. The prolonged stays that many patients with COVID-19 experience also promotes stasis, and therefore also thromboembolic phenomena [71,72]. Evidence shows that COVID-19 patients have a prevalence of about 25% of ultrasound-confirmed, deep venous thrombosis [73,74].

## 3. Materials and Methods 

This work started as a literature review, but developed into a position statement, bringing together the opinions of authors who have been involved in cardiac rehabilitation for years. The references are based on studies conducted on non-COVID patients with cardiovascular disease.

Papers were identified via a search of PubMed, Scopus, and Pedro databases, in order to highlight the cardiovascular complications of COVID-19 and to define the physiotherapy treatment recommended for these patients. Literature search terms included ‘Coronavirus’, ‘COVID-19’, ‘severe acute respiratory syndrome/SARS’, ‘Middle Eastern respiratory syndrome/MERS’ and were combined in multiple strings using the Boolean operator AND with the following terms ‘Cardiac rehabilitation’, ‘Recovery’, ‘Cardiovascular complications’, ‘Exercise training’, ‘Exercise dose’, ‘Cardiac telerehabilitation’, ‘Cardiac remote rehabilitation’, ‘Physiotherapy’. These key terms were used in PubMed, Scopus, and Pedro databases and analyzed for literature from the last 15 years. Papers were identified with relevant titles and abstracts reviewed. Literature review revealed a limited number of studies about the rehabilitation of cardiovascular consequences from COVID-19, therefore, this work was developed as a position statement.

## 4. Cardiac Rehabilitation after COVID-19: Position Statement

### 4.1. Exercise Training in the Post-Acute Phase

In both hospital and home settings, it is useful to divide exercise programs into three levels of effort (low, medium, and high) [75,76], based on the patient’s condition. A complete initial assessment should include exercise capacity through the 6 Minute Walking Test (6MWT) [77], physical function through the short physical performance battery (SPPB), strength, and also identify existing impairments in basic activities of daily living (ADL) and instrumental activities of daily living (IADL) [78,79]. The following parameters should be constantly evaluated during the exercise [80,81]:

- Saturation: must remain above 92–93% during the whole exercise [81]
- Heart rate: must not increase more than 20 beats per minute from the baseline heart rate during mild intensity exercise (patient’s pharmacological therapy should also be carefully considered, especially the use of beta-blockers that limit the physiological increase in frequency during exercise) [81]
- Systolic blood pressure: must be ≥90 mmHg and ≤180 mmHg [81]
- Symptomatology: with use of the Borg scale for dyspnea (must not exceed a score of 4) and of the rate of perceived exertion (RPE) scale for fatigue (must not exceed a score of 11–12) [80,81].

The purpose of physiotherapy in the context of cardiovascular complications of COVID-19 is to trigger the systemic antioxidant response to modulate the inflammatory state generated by the virus, and to intervene in the endothelial dysfunction caused by the same. This can be achieved through exercise training, among which the most used types are:

Aerobic endurance training (Table 1, Point a): provides prolonged training periods lasting at least 20 minutes at sub-maximal intensity from 40–60% of the maximum heart rate reserve (HRR), which can be increased up to 80% based on the patient’s condition [81,82], with a frequency of 3 to 5 times per week. It is now established that regular moderate-intensity aerobic exercise increases dependent endothelial vasodilation in subjects with impaired endothelial function, increasing the bioavailability of NO [83,84,85,86]. The effects of the exercise include the activation of systemic antioxidant mechanisms and anti-inflammatory defenses that induce a decrease of arterial stiffness [87,88], with endothelium-dependent vasodilation induced by NO, and therefore, dose-dependent hypotensive effects [89] in terms of extent and duration [90,91,92].

Interval training (Table 1, Point b): interval exercises alternate training periods with periods of rest and can be carried out at various levels of intensity. As a first approach for more compromised post COVID-19 patients, interval training is preferable and better tolerated at an intensity of 2–3 METs, with a frequency of 3 to 5 times a week. Interval exercises, according to some authors, seem to be responsible for cardiovascular changes and endothelial function, in equal or even greater measure than endurance training [92,93,94]. 

In the context of interval training, however, there is a great deal of evidence in favor of the cardiovascular benefits of high intensity interval training (HIIT) (Table 1, Point c). HIIT alternates periods of short and intense anaerobic exercise with periods of recovery with less intense aerobic activity. This variation within the same exercise is responsible for improving endothelial function [95,96], however, in patients post COVID-19, high intensity exercises can only be administered after a careful initial evaluation, and in the post-acute phase for a high level of fatigue and respiratory distress with a frequency of 2 to 3 times a week. 

Resistance training (Table 1, Point d): is an anaerobic exercise mode characterized by the presence of an external load, or the body weight itself. Typically, it is more used in the treatment of sarcopenia [97] than for cardiovascular pathologies. We can distinguish two kinds of training: resistance training, which involves specific muscle groups, and circuit training, which includes the whole body, thus generating a more important hypotensive response [98]. As part of the cardiac rehabilitation programs for post COVID patients, resistance training should be offered at moderate intensity equal to 8–12 repetition maximum [81] at a frequency of 2–3 times a week. However, intensity and frequency, as in the case of HIIT must be modulated in relation to the clinical and hemodynamic conditions of the patients [99]. From a cardiovascular point of view, resistance training during exercise is accompanied by significant increases in blood pressure and heart rate [100]. At the same time, there is a reduction in post-exercise pressure that lasts up to 24 hours [98]. In a study conducted on hypertensive rats, it appears to have induced improvements in endothelial function mediated by an increase in NO, together with a reduction in systemic inflammation [101], even though these results are considered to be of lesser extent than endurance training [102].

Despite the low incidence of adverse events during cardiac rehabilitation, in post-COVID patients it is appropriate to keep in mind the following elements, which require further study, and a possible suspension of the physiotherapy [81,103,104]:

- Saturation <88–93%
- Heart rate <40 beats per minute, or >120 beats per minute
- Systolic blood pressure <90 mmHg and >180 mmHg
- Body temperature fluctuations >37.2 °C
- Respiratory symptoms and fatigue that worsen during exercise and are not alleviated after rest
- Symptoms such as chest tightness or pain, difficulty in breathing, severe cough, dizziness, headache, unclear vision, palpitations, sweating and instability.

These parameters must be targeted to the specific risk profile of the patient, according to the response obtained at the 6MWT performed during the physiotherapy evaluation.

Exercise training is therefore a powerful tool in physiotherapy that is capable of inducing significant changes in the cardiovascular system and functional to the recovery of the endothelial dysfunction, which is now recognized as responsible for numerous pathologies [105]. Evidence highlights the clinical outcomes of cardiac rehabilitation on endothelium and myocardium in patients with acute myocardial infarction or who have undergone coronary artery bypass graft surgery (CABG) surgery, percutaneous coronary intervention (PCI), heart transplantation, heart valve surgery, and in patients with chronic heart failure (CHF) [106]. In particular, clinical effects of exercise have been reported on coronary endothelial function in patients with coronary artery disease (CAD) [107], demonstrating that 4-weeks of exercise training was effective in attenuating the paradoxical arterial vasoconstriction in epicardial conduit vessels by −54% and increasing average peak flow velocity by +78%. In addition, Belardinelli and colleagues [108], who performed an exercise training program in heart failure (HF) patients for >10 years, demonstrated an improvement in quality of life and a reduction in major cardiovascular events, including hospitalizations for chronic heart failure and cardiac mortality. Finally, Ades et al. [109] have clearly reported the clinical outcomes that can be obtained by a cardiac rehabilitation program, classifying them as: (1) primary clinical outcomes, (2) intermediate clinical outcomes, (3) quality-of-life, and they defined the improvement measurable at different levels: cardiovascular, metabolic, skeletal muscle and psychologic [109]. 

Due to the wide variety of possible exercise programs that can be obtained by combining intensity, duration, speed of execution, and exercise mode in various ways, and defining the program on the basis of constant patient monitoring, exercise training is well suited to the treatment of post-COVID patients with impaired cardiovascular system of various degrees.

### 4.2. Exercise Dose and Adverse Effects

Based on the data reported so far, it is necessary to underline the importance of the exercise dose that is proposed to our patients since exercise acts as a real biological drug that responds to the principle of hormesis, according to which, it is the dose that determines the beneficial or harmful effects, depending on the size of the stimulus [110]. This transition threshold could be influenced by demographic characteristics such as age, gender, ethnicity, and primary risk for cardiovascular diseases [111]. Moreover, before starting any kind of exercise training, it is important to follow the recommendations in the European Society of Cardiology (ESC) guidelines [112,113], which suggest avoiding exercise after viral myocarditis and cardiomyopathy for 6 months.

Some studies conducted on endurance runners have reported that exercise at high doses and for long periods can induce effects such as pathological remodeling of the heart and large vessels, a transient volumetric overload of the atria and of the right ventricle with transient reductions in the ejection fraction and coronary calcification [114]. Practicing three times more exercise than the recommendations in the guidelines, and for more than 25 years also appears to predispose individuals to subclinical coronary atherosclerosis, although this result can only be related to Caucasians and males [115].

In a study conducted on marathon runners who have run at least one marathon per year for 25 years, an increase in coronary artery calcification was noted, however it appeared to be related to the presence of cardiovascular risk factors and not to the number of marathons or training years [116]. Two other studies investigating the correlation between high exercise levels and coronary calcification have found a higher presence of atherosclerotic plaques in highly trained subjects compared to sedentary ones, however, these have better stability and a more favorable composition for the same cardiovascular risk profile [117,118]. In conclusion, the association between exercise and a higher survival is now certain, but the right dose for each individual and how it changes based on parameters such as age, gender and cardiovascular risk profile is not yet known. From a translational standpoint, in the analysis of the effects of long-term exercise, it could be useful to combine this with an in vitro study of the molecular mechanisms that underlie the cardiovascular effects of exercise in humans [119]. It is well-known that a virus binds and enters through ACE2, affecting systemic inflammation, multiorgan dysfunction, and the cardiovascular system, and leading to several complications including myocardial injury, myocarditis, acute myocardial infarction, heart failure, dysrhythmias, and venous thromboembolic events. 

Considering the beneficial effects of long-term clinical rehabilitation and exercise established by decades of research in patients with CVDs [120,121,122,123], we can hypothesize that a cardiac rehabilitation program designed to meet the specific patient’s needs may be helpful to reduce complications and mortality in patients with COVID-19 and cardiovascular diseases.

The long-term benefits of exercise in the cardiovascular patient are proportional to the weekly exercise time and chronic training [124].

### 4.3. Respiratory Rehabilitation

Exercise training in COVID-19 patients is almost always characterized by respiratory problems of various degrees. Thus, performing respiratory rehabilitation in COVID-19 patients helps to ameliorate dyspnea, alleviate anxiety and depression, reduce complications, prevent and improve dysfunction, reduce morbidity, preserve functions, and improving quality of life is imperative. Clearly, to pursue this aim, it is important to follow the recent recommendations for respiratory rehabilitation in adults with COVID-19 [103,125]. For severe and critically ill patients with unstable and progressive deterioration, early respiratory rehabilitation is not recommended in order to exclude complications and not aggravate the burden of infection. In contrast, after examination of each patient’s condition, evaluating his systemic function, particularly in terms of cognitive status, respiratory function, cardiovascular function, and musculoskeletal function, the respiratory rehabilitation program should be customized on the basis of the unique problems of each patient. 

The main goals of post-acute treatment are:

Improvement of ventilation of the deep lung: Using volume strategies including chest expansion exercises (thoracic expansion exercises, TEE). These focus on inspiration and are characterized by deep and slow breaths up to the volume of the inspiratory reserve, an end of inspiration pause, followed by an unforced expiration up to residual functional capacity [126,127].

We recommend four to five repetitions to avoid hyperventilation [128].

The purpose of this technique is to activate collateral ventilation, improve the distribution of the inspired air, bring air behind the secretions, and reduce the resistance of the airways.

Airway clearance: Using techniques that exploit positive expiratory pressure (PEP), this is recommended only in the post-acute phase in stabilized patients, especially those with previous chronic obstructive respiratory diseases [125]. The use of PEP in patients with acute respiratory failure is to be avoided because it could expose them to an increase in respiratory distress [129]. For airway clearance, it is also useful to improve cough effectiveness with the use of aids such as cough machines, but avoiding tiring the patient too much or causing symptoms such as dyspnea, pain, or chest tightness [81].

Respiratory rehabilitation in patients with highly transmissible infectious diseases, such as COVID-19, must be done while paying great attention to the risk of contagion for the physiotherapists who deal with it, as all these procedures are potentially dangerous for the production of aerosols [130]. In this regard, the proper use of personal protective equipment (PPE) [131] is strongly recommended.

### 4.4. Exercise Training In-Home Settings: Telerehabilitation

During the emergency linked to the spread of Sars-Cov-2, physiotherapy intervention requires remodulation to guarantee the patient recovers their health, and at the same time, to protect the physiotherapist against the risk of contagion. In addition, it is necessary to consider the restrictions imposed by the authorities to prevent the spread of the infection, which cause increasing difficulties in providing rehabilitation assistance in outpatient and home settings, and it is also necessary to lighten the burden of acute care by transferring post-COVID patients to rehabilitative structures [132]. When the rehabilitation intervention cannot be carried out in direct contact with the patient, telerehabilitation may be helpful as an alternative strategy; this involves the use of video calls or adequately structured platforms.

In cardiac rehabilitation, there are already promising experiences described in the literature that provide for the use of tele-rehabilitation for a higher number of patients and for a favorable cost/effectiveness ratio [133,134]. Remote cardio-rehabilitation is safe and effective even for patients with cardiovascular disease or post cardiac surgery [135]. However, monitoring systems that provide for oximetry [136], blood pressure control are required [136], as well as electrocardiography, especially in the management of complex patients [137,138]. It is useful to correlate the administration of the Borg scale for dyspnea and rate of perceived exertion (RPE) [139] with the detection of vital signs.

Cardiac telerehabilitation is mainly based on exercise training in interval or endurance mode, with calisthenics exercises or with the use of a cycle-ergometer or treadmill. The intensity of the exercise is established for each patient on the basis of the initial assessment, the hemodynamic parameters assessed remotely [132] with devices such as the oximeter and telemetry, and the symptoms investigated with the administration of scales [137], such as the Borg scale for dyspnea and RPE. Exercise training should also include counseling strategies, patient education, psychological support [139] and nutritional interventions [140]. A form of hybrid treatment may be appropriate for this type of patient, limiting in-presence physiotherapy to a minimum, preferring the remote modality and scheduling periodic evaluations and treatments in-presence [135].

## 5. Limitations

We were able to evaluate a limited amount of literature, considering the recent onset of the disease and considering that in the first months of the pandemic, all human and material resources were invested in the quoad vitam prognosis of patients with COVID-19 and not on the rehabilitation process of affected patients. However, now it is possible to conduct studies on the post-acute rehabilitation of post-COVID patients and to investigate their follow-up with subsequent studies.

## 6. Conclusions

Owing to the recent onset of the disease, evidence concerning the rehabilitation treatment of cardiovascular complications from COVID-19 is scarce in the literature. The primum movens of cardiovascular complications seems to be endothelial dysfunction [141,142], and also connected to the severe thromboembolic complications at venous, pulmonary and cerebral level that have been recorded in many patients [142,143,144], including also young patients [145]. Therefore, in the present work, we tried to set up an exercise program aimed at improving endothelial function. Of great importance is a multidisciplinary approach involving exercise, diet, and psychological support for the correction of cardiovascular risk factors [139,140]. Moreover, it is important to keep in mind that, although the exercise exerts beneficial effects on endothelial function as evidenced in acute myocardial infarction, CAD, and HF patients and contributes to the reduction of cardiovascular alterations [146], whether it actually translates into improved clinical outcomes in COVID-19 patients remains to be demonstrated. 

Thus, any patient with COVID-19 would require a complete assessment of their symptoms, exercise capacity, function, and potential impairments [81]. Depending on the patient’s initial assessment and their clinical and cardiovascular risk profile, an exercise program should be developed that considers all clinical features of the patient. Exercise is considered to be a biological drug [110], so we must pay close attention to ensure the right dose is administered to our patients. Exercise can be modulated in terms of intensity, frequency and speed of execution in order to adapt programs to this novel group of patients that is emerging as a result of the COVID-19 pandemic.

## Figures and Tables

**Table 1:** Exercise training and cardiovascular effects. Abbreviations: HRR, heart rate reserve; RPE, rate of perceived exertion; NO, nitric oxide; MET, metabolic equivalent of task; RM, repetition maximum.

Training Type	Exercise Description	Exercise Frequency	Cardiovascular Effects
(a) Endurance training(ET)	Aerobic Continuous exercise periods, at least 20 minutes Low-moderate intensity, 40–80% HRR, RPE = 12	3–5 times per week	Endothelium dependent vasodilation Endothelial function improvements Increased bioavailability of NO Activation of systemic antioxidant and anti-inflammatory defenses Decrease in arterial stiffness BP reduction
(b) Interval training(IT)	Aerobic Series of moderate-high intensity exercises, interspersed with rest Intensity 2–3 METs	3–5 times per week	Cardiovascular function improvements Endothelial function improvements (even greater than endurance training)
(c) High Intensity IT(HIIT)	Aerobic-Anaerobic Series of high intensity exercises, interspersed with less intense recovery periods Usually lasts under 30 minutes	2–3 times per week	Endothelial function improvements (linked to the intensity variation within the same exercise)
(d) Resistance training(RT)	Anaerobic Series of 8–12 repetitions, with 2–3 minutes of rest (2–4 sets) Moderate intensity, 8–12 RM Resistance is offered by an external load (e.g., elastic band) or body weight	2–3 times per week	Rapid increases in BP and HR, during the exercise BP reduction that lasts up to 24 h, in post exercise Endothelium dependent vasodilation (<than ET) Endothelial function improvements (<than ET)

## Data Availability

Not applicable.
